# A *Toxoplasma gondii* vaccine encoding multistage antigens in conjunction with ubiquitin confers protective immunity to BALB/c mice against parasite infection

**DOI:** 10.1186/s13071-015-1108-7

**Published:** 2015-09-30

**Authors:** Huiquan Yin, Lingxiao Zhao, Ting Wang, Huaiyu Zhou, Shenyi He, Hua Cong

**Affiliations:** Department of Human Parasitology, Shandong University, School of Medicine, No. 44 Wenhuaxi Road, Jinan, Shandong 250012 PR China

**Keywords:** Adenovirus vaccine, DNA vaccine, Prime-boost strategy, Toxoplasma gondii, Ubiquitin-conjugted

## Abstract

**Background:**

*Toxoplasma gondii* is a widely prevalent intracellular parasite which infects almost all warm-blooded animals including humans and causes serious zoonotic toxoplasmosis. DNA vaccines have proved effective in the protection against parasites. However, the problems of weak immunity and inefficient delivery of DNA vaccine remain major issues. Therefore, comprehensive antigens derived from all stages of the parasite, effective adjuvants and delivery systems should be considered in the vaccine construction.

**Methods:**

SAG3_101–144_,ROP18_347–396_, MIC6_288–347_, GRA7_182–224_, MAG1_58–125_, BAG1_156–211_ andSPA_142–200_, derived from antigens in tachyzoite, bradyzoite and sporozoite stages of *T. gondii* were screened based on CD8^+^ T cell epitope binding affinity to HLA and H-2. We constructed a recombinant DNA vaccine and an adenovirus vaccine encoding multi-stage antigen of *T. gondii* linked to ubiquitin molecules and vaccinated BALB/cmice with different strategies. Antibodies, cytokines, splenocytes proliferation, as well as the percentage of CD4^+^ and CD8^+^ T cells in immunized mouse were analyzed by the Enzyme-Linked Immunosorbent Assays (ELISA), Flow Cytometry (FCM). Protective efficacy was evaluated by challenging immunized mice with type I and type II parasite.

**Results:**

Our results indicated that the DNA vaccine had the advantage of inducing a stronger humoral response, whereas the adenovirus-vectored vaccine effectively improved the cellular immune response. Priming with DNA vaccine and boosting with adenovirus-vectored vaccine induced Th1-type immune responses with highest levels of IgG2a and secretion of cytokines IL-2 and IFN-γ. Effective protection against type I and type II parasite with an increase in survival rate and a decrease in brain cyst burden was achieved in immunized mice.

**Conclusions:**

Priming vaccination with DNA vaccine and boosting with the recombinant adenovirus vaccine encoding ubiquitin conjugated multi-stage antigens of *T. gondii* was proved to be a potential strategy against the infection of type I and type II parasite.

**Electronic supplementary material:**

The online version of this article (doi:10.1186/s13071-015-1108-7) contains supplementary material, which is available to authorized users.

## Background

Toxoplasmosis, a zoonotic disease transmitted between different host species, can infect all warm blooded mammals and birds worldwide [1–3]. An acute infection with this parasite in the early stage of pregnancy can cause prenatal malformations and abortion in hosts [4]. The parasite cyst in the muscle of animals and the oocyst in the feces of cats pose a potential threat to human health.

Many efforts have been made to develop vaccines against *T. gondii* including killed vaccines, live attenuated vaccines, and genetic engineering vaccines. The only licensed vaccine “TOXOVAX” for veterinary use is based on S48 strain which is a live attenuated vaccine. However, this kind of vaccine poses a risk of infection to human and animals handling the vaccines for the reason of virulence restoration. Numerous studies of preventive immunization in mice have exploited the conventional *T. gondii* antigen-based DNA vaccines [5–7]. However, vaccines based on antigens expressed in the single stage can’t induce complete protective immunity against *T. gondii* [8, 9]*.* The complex life cycle of *T. gondi* has three major infectious stages: tachyzoites, bradyzoites (in tissue cysts) and sporozoites (in oocysts). A vaccine containing antigens derived from all stages of the parasite life cycle is required. The vaccine induction of potent, long-lived CD8^+^ T cells has become a major goal of current *T. gondii* vaccine efforts [10–12]. It is preferable to construct antigen segments derived from antigens that contain specific CD8^+^ T cell epitopes from the different life cycle stages.

Effective adjuvants and delivery systems were considered to construct an effective *T. gondii* vaccine. Ubiquitin, a 76-amino-acid peptide, has been reported to enhance DNA vaccine responses towards antigens in the adjuvant setting [13, 14]. Conjugating ubiquitin to a DNA construct was intended to enhance the proteasome dependent degradation of endogenously synthesized antigens, which would result in an increased cell-mediated response against the conjugated antigen *in vivo* [15–17]. However, how to raise the transfection efficiency of DNA vaccine into immune cells is still a problem. Some studies have suggested that using adenovirus serotype 5 (Ad5), a replication-defective adenovirus serotype, as the vaccine vector could elicit vigorous and sustained T-cell responses [18, 19]. Vaccine studies on Ebola virus [20], HIV [21] and the malaria parasite [22] have proved recombinant adenovirus-based vaccine could elicit antibodies, T-cell responses and provide long-term protection. Clinical trials on HIV and tuberculosis have shown that vaccines based on Ad5 are safe and highly immunogenic [23, 24]

Therefore, in this study, SAG3_101–144_, ROP18_347–396_, MIC6_288–347_, GRA7_182–224_, MAG1_58–125_, BAG1_156–211_ and SPA_142–200_, derived from antigens in tachyzoite, bradyzoite and sporozoite stages of *T. gondii* were screened based on CD8^+^ T cell epitope binding to HLA and H-2 restricted. The immune response and protection efficacy was evaluated via inoculation of BALB/c mice with DNA vaccine or/and adenovirus vaccine encoding ubiquitin-conjugated multistage antigens of *T. gondii*.

## Methods

### Mice

Specific-pathogen-free female BALB/c mice (6 to 8 weeks old) were purchased from Shandong University Laboratory Animal Centre (Jinan, China). Mice were housed 5 per cage under pathogen-free conditions and were adequately supplied with sterilized water and food.

### Ethical approval

All experimental procedures with animals used in the present study had been given prior approval by the Institutional Animal Care and Use Committee of Shandong University under Contract LL2015-02. Humane endpoints are chosen to terminate the pain or distress of the experimental animals via euthanasia. Mice were monitored daily over 8 weeks for signs of toxoplasmosis including food and water intake difficulties, fatigue, severe ascites, any that showed signs of illness were sacrificed immediately with CO_2_ gas.

### Parasites

*T. gondii* strains, RH strain (type I) and PRU strain (type II) were used for *in vivo* challenges in this work. Tachyzoites were created, maintained and utilized as previously described [25]. Briefly, parasites were cultured in Dulbecco’s modified Eagle’s medium supplemented with 10 % fetal calf serum, penicillin (100 U/ml), streptomycin (100μg/ml), and L-glutamine (2mM) in a humidified incubator at 37°C with 5 % CO_2_ and maintained by passage in HeLa cells.

### Antigen and peptides screening

Bioinformatic algorithms from the Immune Epitope Database, http://www.iedb.org/ were used to predict CD8^+^ T cell epitope conserved regions. Protein fragments SAG3_101–144_, ROP18_347–396_, MIC6_288–347_, GRA7_182–224_, MAG1_58–125_, BAG1_156–211_ and SPA_142–200_ were screened based on their predicted binding affinity to HLA (HLA-A*02, HLA-A*03 and HLA-B*07) and H2 (H2-Ld, H2-Dd and H2-Kd) supertype molecules; those with a percentile rank lower than 50 were selected (Additional file 1: Table S1).

Peptides ( >95 % purity) (p1 to p7) derived from above protein fragments with high binding affinity were synthesized by ChinaPeptides (Shanghai, China) and stored at–80°C until the candidate vaccine is analyzed.

### Construction of the recombinant DNA vaccine and adenovirus vaccine

The compound genes encoding multistage antigen segments (MAS) and ubiquitin-conjugated multistage antigen segments (UMAS) of *T. gondii* were designed by linking the selected protein segments with the spacer sequence Ala-Ala-Tyr and synthesized by GENEWIZ (Suzhou, China). The genes encoding MAS or UMAS were cloned into the EcoRI/XbaI or BamHI/XbaI site of eukaryotic expression plasmid pVAX1. Recombinant plasmids ((p-MAS or p-UMAS) were purified by an endotoxin-free plasmid purification kit (Cwbio, China).

**Fig. 1:**
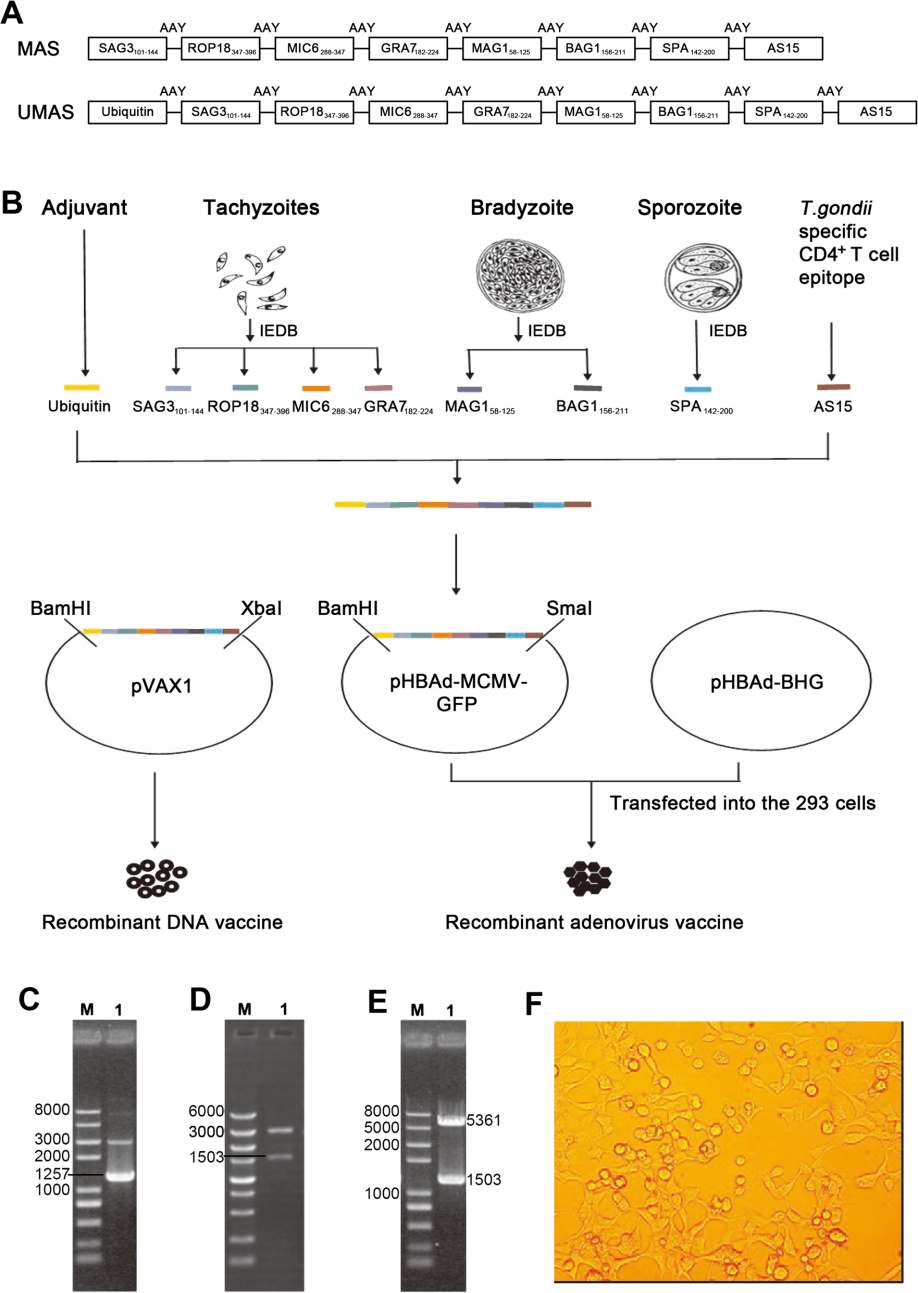
Construction of DNA and adenovirus vaccines encoding ubiquitin-conjugated multistage antigen segments derived from *T. gondii*. **a** Schematic representation of the compound proteins, which encode amino acid sequences of eight protein segments derived from tachyzoite, bradyzoite and sporozoite antigens of *T. gondii*. **b** Construction of recombinant DNA and adenovirus vaccines expressing ubiquitin-conjugated multistage antigen segments. The recombinant eukaryotic plasmids pVAX1-MAS (**c**), pVAX1-UMAS (**d**), and pHBAd-MCMV-GFP-UMSA (**e**) were identified by restriction enzyme digestion analysis (Lane 1). Lane M: DNA marker. **f** Recombinant adenoviral particles were obtained from 293 cells

Recombinant adenovirus expressing UMAS (Ad-UMAS) was generated using the AdMax^TM system (Hanbio, Shanghai, China) by homologous recombination of pHBAd-MCMV-GFP-UMAS with pHBAd-BHG in HEK-293 cells. The titre of 10^11^ plaque-forming units (PFU)/mL Ad-UMAS particles was purified by cesium chloride gradient centrifugation and then stored in storage buffer (10 mM Tris, 2 mM MgCl_2_, 5 % sucrose, pH 8.0) at–80°C. Figure 1 shows the construction of the DNA vaccines (p-MAS and p-UMAS) and adenovirus vaccine (Ad-UMAS).

### Mice immunization and challenge study

Eight groups BALB/c mice with 15 mice per group were immunized twice via intramuscular injection at three-week intervals. Mice were vaccinated with the DNA vaccines (p-MAS or p-UMAS plasmid; 100 μg each) or recombinant adenovirus vaccine (Ad-UMAS virus, 3 × 10^8^ PFU each) or the combination of DNA vaccine (p-UMAS, 100 μg each) and recombinant adenovirus vaccine (Ad-UMAS virus, 3 × 10^8^ PFU each). Mice injected with PBS (100 μL each), pVAX1 (100 μg each) or Ad-GFP (an empty adenovirus; 3 × 10^8^ PFU each) were served as control group separately.

Four weeks after the boost immunization, immunized mice were challenged with the type I or type II parasites to evaluate their abilities against infection. Five immunized mice per group were infected with 1 × 10^3^ tachyzoites of *T. gondii* RH strain (type I parasite) and the survival time of the mice was observed and recorded. Another six immunized mice per group were infected via the gastric route with 20 cysts of *T. gondii* PRU strain (type II parasite) and the cyst burden in the mice brains was counted four weeks later. The mean number of cysts per brain was determined by counting three samples of 10 μL aliquots from each homogenized brain in 1 mL PBS under an optical microscope.

### Measurement of humoral response

Serum samples were collected by retro-orbital bleeding on days 0, 14, 35 and 49. Standard ELISAs were used to determine the levels of *T. gondii*-specific antibodies, IgG, IgG1 and IgG2a, in the serum samples from five inoculated mice from each group as described previously [26]. Briefly, a flat-bottom 96-well plate was pre-coated with the peptide pool (p1 to p7) at a concentration of 10 μg/mLin a 50 mM carbonate-bicarbonate buffer (pH 9.6) overnight at 4°C. The mouse serum were diluted in PBS (1:100) and horseradish peroxidase–conjugated goat anti-mouse IgG, IgG1 or IgG2a (Sigma-Aldrich, USA) were used as the secondary antibody to detect bound antibodies. The optical density was read at 450 nm in a Thermo Scientific Multiskan FC Microplate Photometer (Thermo Scientific, USA).

### Splenocytes proliferation assay

Four weeks after the final immunization, spleens were removed from three immunized mice per group. Isolated splenocytes were plated in 96-well plates, at a density of 1 × 10^6^ per well, in 100 μL RPMI-1640 medium (Sigma-Aldrich, USA) supplemented with 10 % fetal calf serum and cultured with peptide pools p1 to p7 (10 μg/mL each) or not. Cell proliferative activity was measured following the manufacturer’s instructions on a Dojindo Cell Counting Kit-8 (Dojindo, Japan). The results were expressed as absorbance at 450 nm.

### Cytokines production

The levels of cytokines production were determined using splenocytes from three immunized mice per group four weeks after the final immunization. The splenocytes were cultured with peptide pools p1 to p7 (10 μg/mL each) in 12-well plates at 37°C in 5 % CO_2_ and supernatants were harvested and assayed for IL-2 at 24 h, IL-10 at 72 h, and IFN-γ at 96 h using a commercial ELISA Kit (R&D Systems, USA), following the procedure recommended by the manufacturer.

### Murine splenic T lymphocytes staining

Splenocytes from three immunized mice separated as described above were washed with PBS and then incubated with a 1:1000 dilution of anti-mouse CD8a-polyethyleneimine and CD4-fluorescein isothiocyanate T cell antibodies (eBioscience, USA) at 4°C for 30 min. After washing, cells were resuspended in PBS and analyzed for CD8^+^ T cells and CD4^+^ T cells by Flow Cytometer (Beckman Coulter FC500, USA).

### Statistical analysis

SPSS 19.0 software was used in the statistical analysis. Differences between the groups were tested by analysis of unpaired student’s t-test. Survival rate was compared by the Kaplan–Meier method. The difference was considered significant if *p-*value was less than 0.05.

## Results

### DNA vaccine encoding ubiquitin-conjugated multistage antigen segments induced a specific immune response in mice

**Fig. 2:**
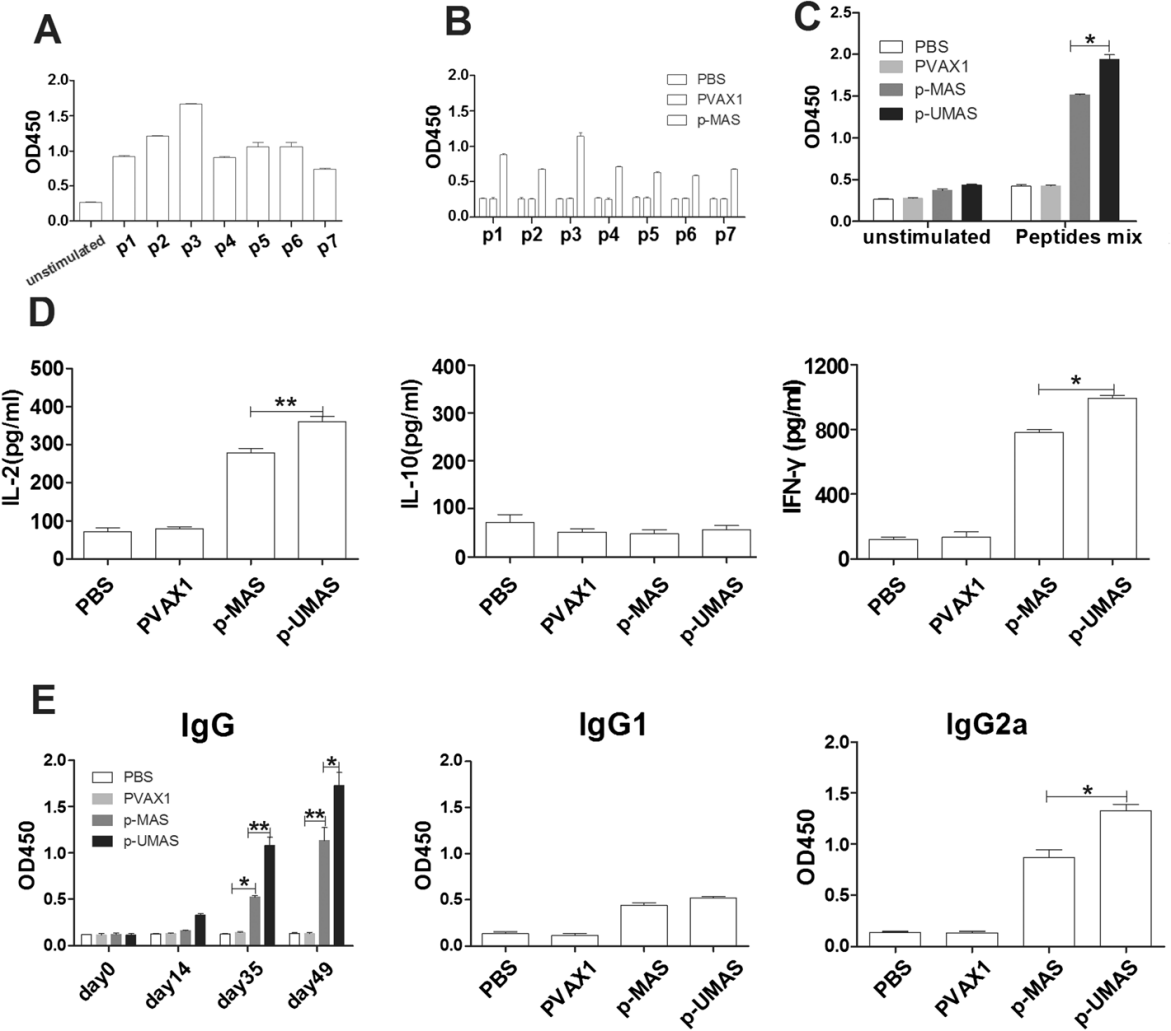
DNA vaccine encoding ubiquitin-conjugated multistage antigen induced a specific immune response in BALB/c mice. p-UMAS and p-MAS encoding *T. gondii* multistage antigens with or without ubiquitin adjuvant immunized BALB/c mice twice at three-week intervals. Levels of splenocyte proliferation in mice are presented as mean optical density at 450 nm ± SD by stimulation with individual peptide or peptides pool from *T. gondii* infected mice (**a**), DNA vaccine immunized mice (**b**, **c**) through WST-8 assay. **d** Cytokine levels in splenocytes supertanants obtained from immunized mice by culture with peptides pools for IL-2 at 24 h, IL-10 at 72 h, and IFN-γ at 96 h using a commercial ELISA Kit. **e** Antibodies (IgG, IgG and IgG2a) detected in immunized mice serum collected on day 0, 14, 35 and 49 by ELISA. *indicates statistically significant differences between p-UMAS vaccinated mice and p-MAS vaccinated mice

**Fig. 3:**
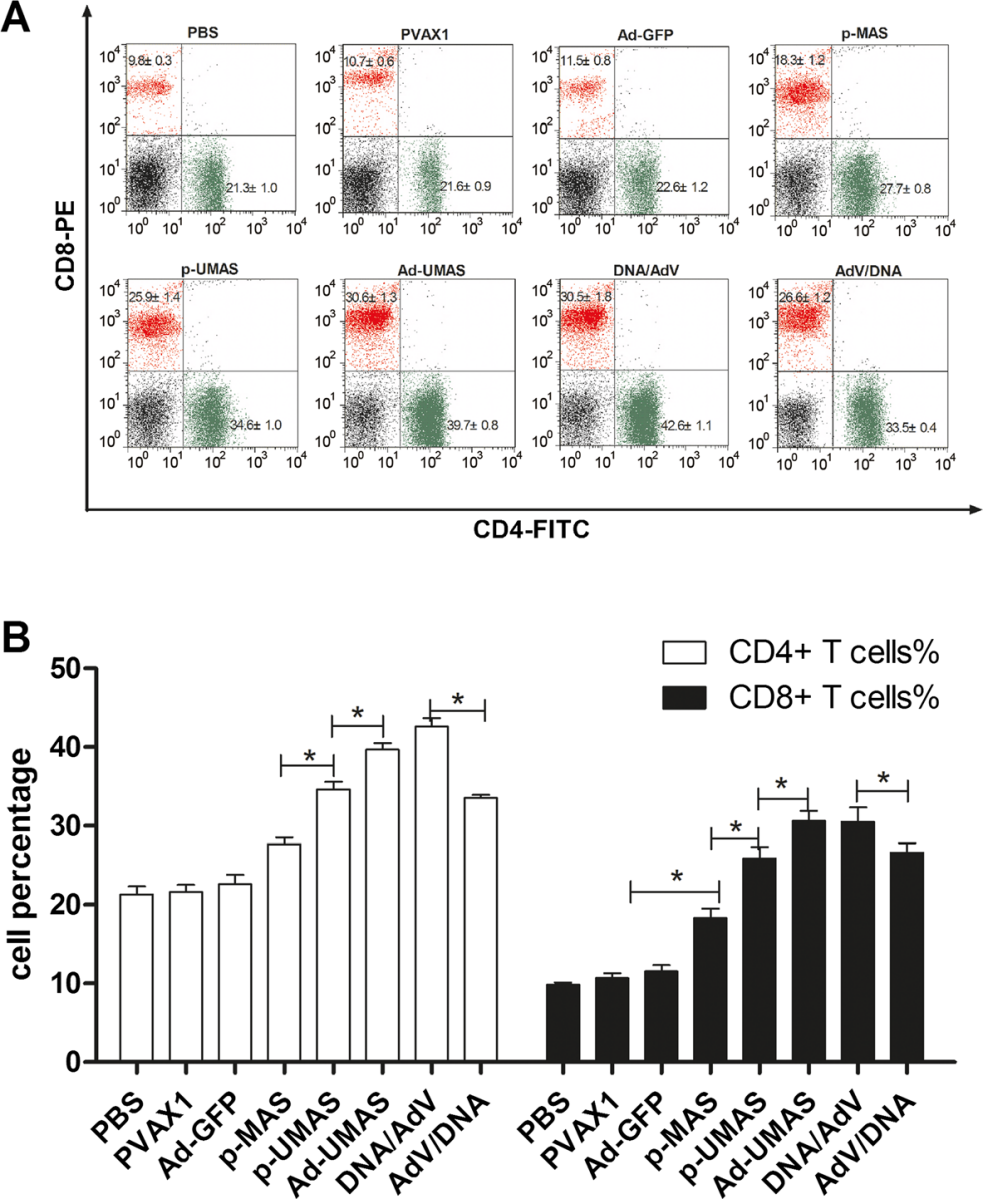
The assay of subtype of T cells by FCM from immunized mice. Four weeks after the last immunization splenocytes from mice were collected and stained with anti-CD8a-PE monoclonal antibodies and anti-CD4-FITC antibodies. Percentage of positive stained cells were analyzed by FCM (**a**, **b**). *indicates statistically significant differences between the marked group

DNA vaccines encoding *T. gondii* multistage antigens with or without ubiquitin adjuvant immunized BALB/c mice twice via intramuscular injection at three-week intervals. The results show that not only specific sera antibodies but also splenocytes proliferation and the secretion of IFN-γ and IL-2 were stimulated in mice immunized with multistage DNA vaccine (p-MAS) (Fig. 2). A further 30 % increase in splenocytes proliferation and higher levels of IFN-γ (991 ± 10.14 pg/mL), IL-2 (360 ± 8.05 pg/mL) were observed in p-UMAS immunized mice than in p-MAS vaccinated mice. Moreover, the level of IgG, predominantly IgG2a, in the serum of p-UMAS vaccinated mice was significantly increased at day 35 and day 49 compared with p-MAS vaccinated mice (*P* <0.05). Furthermore, CD8^+^ T cell percentage were detected greater in the splenocytes of the p-UMAS vaccinated groups than in the p-MAS vaccinated group (*P* <0.05) (Fig. 3). These results indicate that ubiquitin conjugation can effectively improve the immunogenicity of the DNA vaccine and polarize the response towards the Th1-type immune response in mice.

### Adenovirus-vectored vaccine enhanced the cellular immune response in mice

**Fig. 4:**
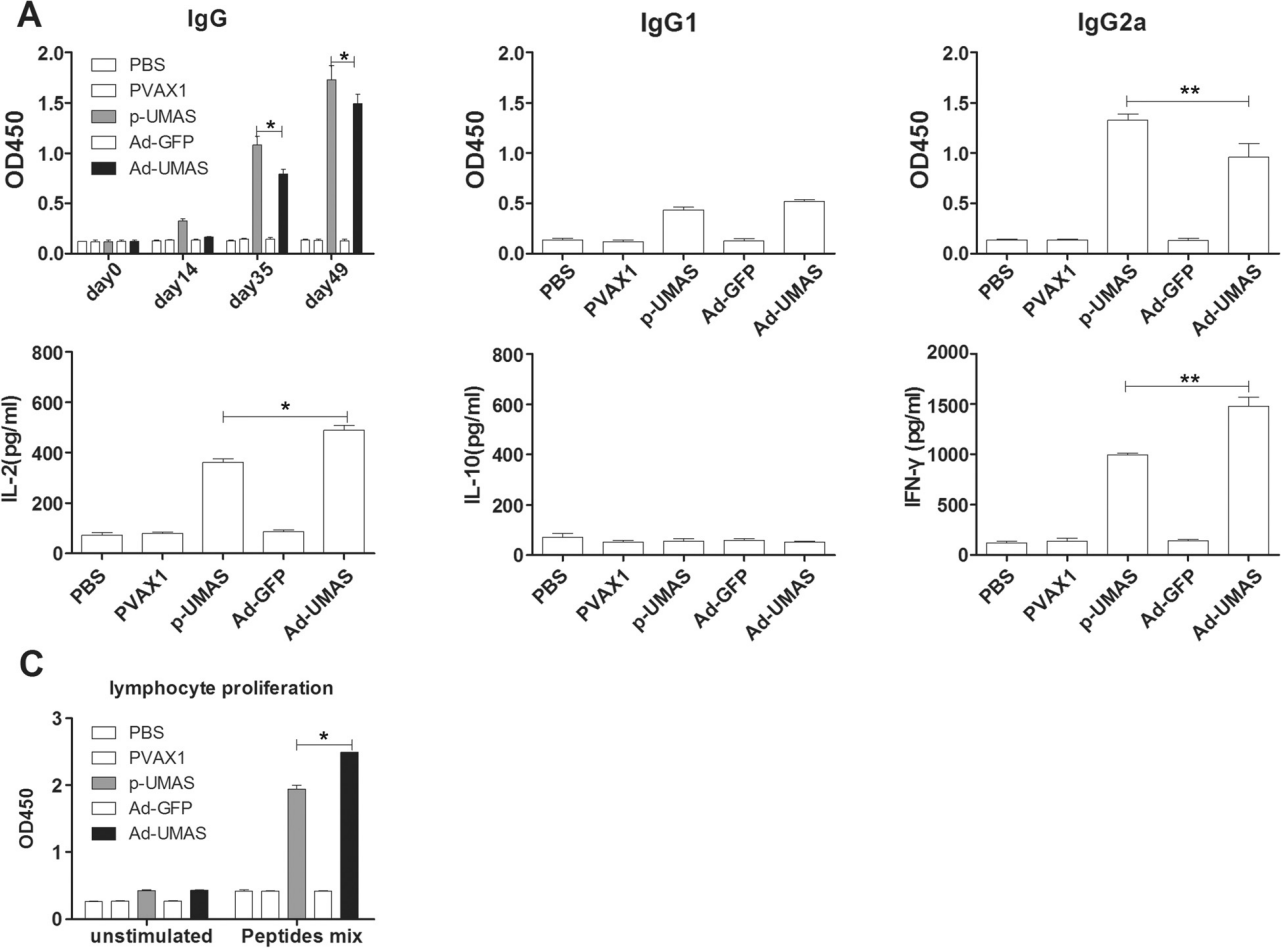
Adenovirus-vectored vaccine enhanced the cellular immune response in BALB/c mice. Ad-UMAS constructed using an adenovirus vector expressing the ubiquitin-conjugated MAS genes. The production levels of antibodies (IgG, IgG1 and IgG2a) (**a**), cytokines (IL-2, IL-10 and IFN-γ) (**b**), and splenocytes proliferation (**c**) were detected same as Fig. 2. *indicates statistically significant differences between Ad-UMAS vaccinated mice and p-UMAS vaccinated mice

The recombinant adenovirus vaccine (Ad-UMAS) was constructed using an adenovirus vector (pHBAd) expressing the ubiquitin-conjugated MAS genes. As shown in Fig. 4, compared with the p-UMAS DNA vaccination group, the levels of IgG, IgG2a in the serum of Ad-UMAS immunized mice was lower, however, significant higher levels of IFN-γ (1478 ± 51.8 pg/mL) and IL-2 (489 ± 11.5 pg/mL) production and enhanced splenocyte proliferation were achieved in the Ad-UMAS vaccine group (*P* <0.05). Notably, the percentages of CD8^+^ T cells in the splenocytes of Ad-UMAS vaccinated mice were significantly augmented compared with p-UMAS vaccinated mice (*P* <0.05) (Fig. 3).

### A robust humoral and cellular immune response was induced by DNA prime–adenovirus boost immunization strategy

To compensate vaccinated mice for the immune response induced by the DNA vaccine and recombinant adenovirus vaccine alone, we investigated vaccination strategy by combining the DNA vaccine (p-UMAS) and recombinant adenovirus vaccine (Ad-UMAS).

**Fig. 5:**
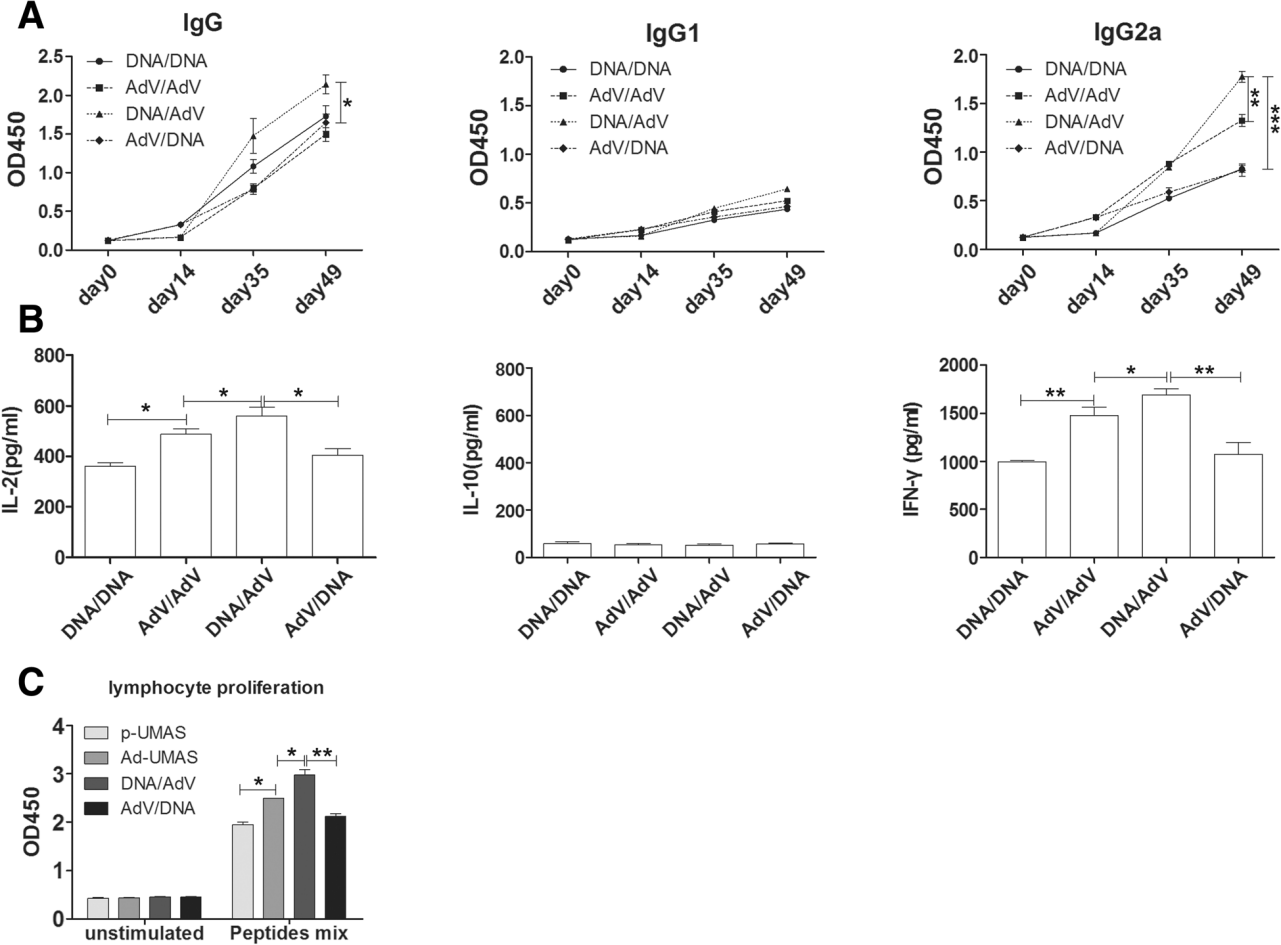
Immune response induced in mice via different vaccination strategies. Mice were vaccinated with the DNA/DNA (p-UMAS plasmid) or AdV/AdV (Ad-UMAS virus) or DNA/AdV (p-UMAS prime, Ad-UMAS boost) or AdV/DNA (Ad-UMAS prime, p-UMAS boost). The production levels of antibodies (IgG, IgG1 and IgG2a) (**a**), cytokines (IL-2, IL-10 and IFN-γ) (**b**), and splenocytes proliferation (**c**) were detected same as Fig. 2. *indicates statistically significant differences between the marked group

As shown in Fig. 5, highest levels of humoral antibodies and cellular immune responses were achieved in mice immunization priming with the DNA vaccine and boosting with the Ad-UMAS vaccine. Compared with p-UMAS or Ad-UMAS immunization alone, higher levels of a specific IgG (predominance of IgG2a) and higher levels of cytokines (IFN-γ, 1691 ± 35.18 pg/mL and IL-2, 561 ± 19.68 pg/mL) were obtained by priming with p-UMAS and boosting with Ad-UMAS (*P* <0.05). When specific CD8^+^ T cell responses for peptides were determined by lymphocyte proliferation activity, priming with p-UMAS and boosting with Ad-UMAS showed the most potent proliferation activity compared with the other immunization strategy (*P* <0.05). However, there was no obvious enhanced immune response when mice received priming with Ad-UMAS and boosting with p-UMAS.

### Protection against type I and type II parasites challenge

**Fig. 6:**
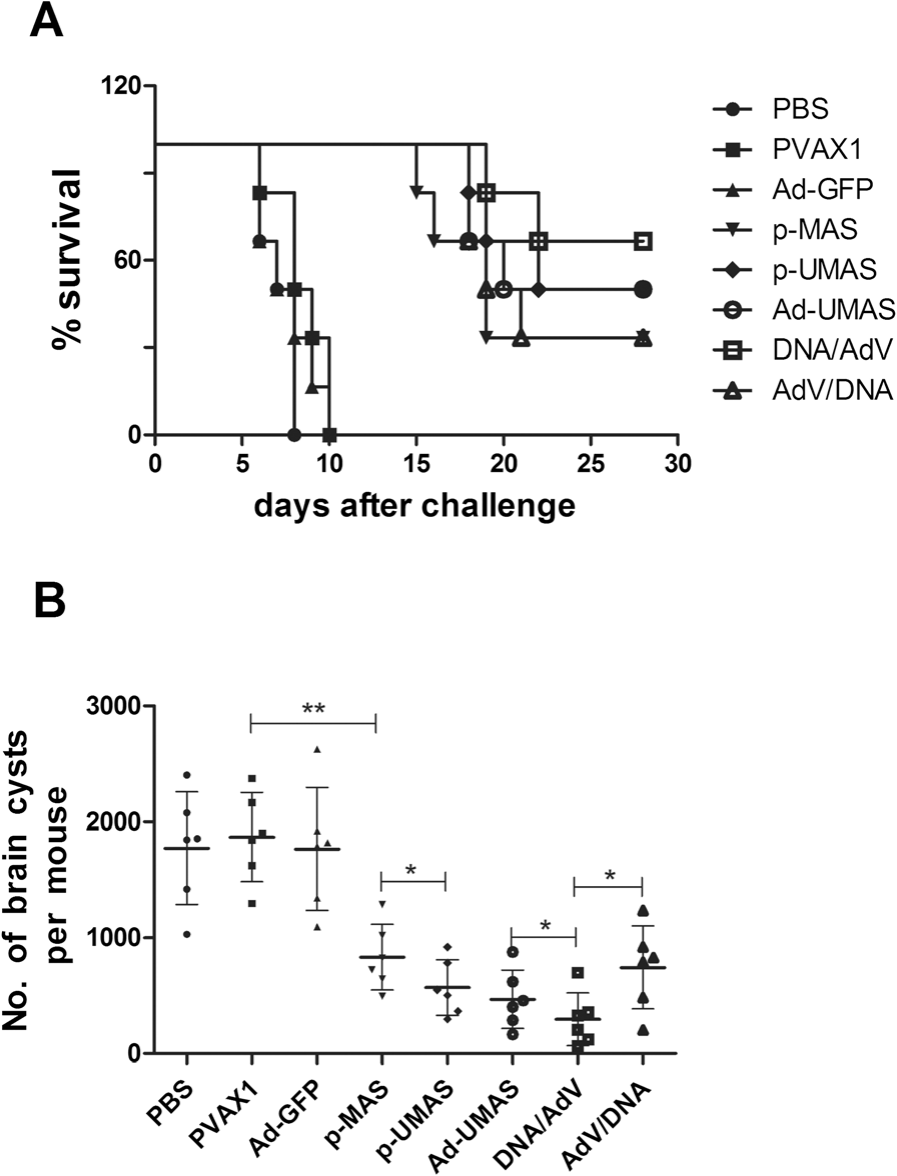
Challenge study of immunized mice against type I and type II parasite infection 4 weeks after the immunization. **a** shows the survival curves of vaccinated mice challenged against the type II parasite challenging. **b** shows the efficiency against the type II parasite challenge by counting the cysts in the brains of immunized mice.*indicates statistically significant differences between the marked group

Figure 6a shows the survival curves of vaccinated mice challenged with 1 × 10^3^ tachyzoites of *T. gondii* RH strain and monitored for 28 days after infection. All the mice in the control groups (PBS, pVAX1, Ad-GFP) died within 10 days. Of the mice vaccinated with the p-MAS DNA vaccine, 33 % survived. The survival rate increased by 17 % when ubiquitin was introduced to the vaccine construction. The highest survival rate, 67 %, was achieved in mice vaccinated with p-UMASprime and Ad-UMASboost .

The efficiency of the vaccine against PRU strain parasite was evaluated by counting cysts in the brains of immunized mice (Fig. 6b). The brain cyst burden in p-MAS immunized mice (833 ± 116) was 50 % lower than that in the control groups (PBS, pVAX1, Ad-GFP) (*P* <0.01). Further decreases in cyst number were detected in the brains of mice immunized with the p-UMAS (570 ± 98) and Ad-UMAS (469 ± 103) vaccines. The most significant reduction of brain cyst burden was achieved by the DNA prime-adenovirus boost approach (296 ± 92).

## Discussion

An ideal vaccine to protect against toxoplasmosis would include more CD8^+^ T cell epitopes, which play a critical role in protective immunity to *T. gondii* in murine models and humans. In this research, seven protein fragments, SAG3_101–144_, ROP18_347–396_, MIC6_288–347_, GRA7_182–224_, MAG1_58–125_, BAG1_156–211_ and SPA_142–200_, derived from antigens in the tachyzoite, bradyzoite and sporozoite stages of *T. gondii* were identified, which contained abounding specific CD8^+^ T cell epitopes. A *T. gondii-*specific CD4^+^ T cell epitope, AS15 [26], was included in the construction for the reason that CD4^+^ T cell help is required in the generation of a CD8^+^ T cell response [27, 28].

In our study, we demonstrate that the DNA vaccine expressing multistage antigen segments of *T. gondii* induced a more robust Th1-prone immunity confirmed by the higher level of IgG2a subtype compared with IgG1. Higher Th1 cytokine levels of IL-2 and IFN-γ, and enhanced CD8^+^ T cells percentage compared with control groups (PBS and pVAX1 immunization groups) showed a strong Th1 immune response. Multistage antigen genes linked by a spacer AAY motif was proved to be easy to bind to a TAP transporter or other chaperons that have a pivotal role in epitope presentation [29].

An ubiquitin gene modified by replacing glycine at the C-terminal with alanine was linked to the N-terminal of the MAS gene to construct the compound p-UMAS DNA vaccine. This modification allowed ubiquitin to remain fused to the protein, resulting in polyubiquitination of the fused non-removable ubiquitin moiety, enhancing the degradation of the substrate protein [30]. The function of ubiquitin in our DNA vaccine construction was investigated on mice inoculation. The level of IgG, predominantly IgG2a, was enhanced when ubiquitin was conjugated. Higher levels of Th1 cytokine IFN-γ, IL-2 and CD8^+^ T cells were also detected in the splenocytes of the p-UMAS vaccinated groups compared to the p-MAS vaccinated group. These data may suggest that the attachment of ubiquitin to a protein could be involved in the protein’s targeted degradation and the production of antigenic peptides presented by MHC class I molecules to CD8^+^ T cells [31]. Previous studies in a porcine circovirus type 2 DNA vaccine also resulted in enhanced cellular and humoral immune responses in immunized BALB/c mice when ubiquitin was incorporated into the vaccine [32].

Compared with DNA vaccine inoculation, mice immunized with recombinant adenovirus vaccine had an enhanced cellular immunity with higher levels of cytokines IFN-γ and IL-2 and a greater percentage of CD8^+^ T cells. These data indicate that the adenovirus-vectored vaccine may effectively improve the cellular immune response, not the humoral response. However, repeated injection of an adenovirus vaccine results in a high titre of serotype rAd5 neutralizing antibody in the immunized animal, which has been suggested to limit the expression of the encoded antigen and dramatically impair the strength of the immune response [33]. Therefore, the alternation of DNA and adenovirus vaccine immunization could be adopted to solve the problem.

Prime-boost vaccination has emerged as an effective strategy for eliciting a robust response to target antigen of *P. falciparum* and *T. gondii* [34, 35]. Even some studies have stated that priming with the viral vector and boosting with a DNA vaccine induces a stronger response [36]. Other studies on vaccines against HBV [37, 38], HCV [39, 40], Ebola virus [41] and influenza virus H5N1 [42] have emphasized that priming with a DNA vaccine and boosting with a recombinant adenovirus vaccine is a much more effective approach. Recently, similar approach with a multiclade HIV-1 DNA plasmid prime and recombinant adenovirus serotype 5 boost has been assessed in both phase I and phase II trials. Promising clinical results showed that the DNA/rAd5 vaccination regimen was safe and induced HIV type 1 multi-clade T cell responses, which were not affected by a pre-existing rAd5 neutralizing antibody titre [43]. In our study,the DNA prime-adenovirus boost immunization strategy resulted in not only a higher level of IgG (IgG2a) but also robust IFN-γ and IL-2 secretion and lymphocyte proliferation. By contrast, priming with Ad-UMAS and boosting with p-UMAS induced an immune response similar to that of the DNA vaccine or adenovirus vaccine alone. Thus indicated priming with a DNA vaccine and boosting with an adenovirus-vectored vaccine resulted in strong Th1-type immune responses and higher protection, with an increase in survival rate and a decrease in brain cyst burden. Although the data we obtained didn’t show 100 % protection in vaccinated mice, our observations provide valuable information for the establishment of an optimal prime-boost vaccination combination of DNA vaccine and adenovirus vaccine against *T. gondii*.

## Conclusions

Distinct humoral and cellular immunity induced by immunization with DNA vaccine and recombinant adenovirus vaccine encoding ubiquitin conjugated multistage antigen of *T. gondii*. The DNA vaccine had the advantage of inducing stronger humoral response, whereas the adenovirus-vectored vaccine improved the cellular immune response. Priming vaccination with the DNA vaccine and boosting with the recombinant adenovirus vaccine was proved to be a potential strategy to protect mice against the infection of *T. gondii*.

## Additional files

Additional file 1: Table S1. The multistage antigen segments selected from *T .gondii*. Antigen fragments derived from tachyzoites, bradyzoies and sprozoites antigens of *T. gondii* were screened based on their predicted binding affinity to HLA (HLA-A*02, HLA-A*03 and HLA-B*07) and H2 (H2-Ld, H2-Dd and H2-Kd) supertype molecules. The epitopes from the antigen segments which have the percentile rank of lower than 50. Peptides (p1 to p7) marked in bold were derived from above protein fragments with high binding affinity and were used for analysis. (DOC 54 kb)
