# Supplementary material for: A Toxoplasma gondii vaccine encoding multistage antigens in conjunction with ubiquitin confers protective immunity to BALB/c mice against parasite infection
Source: Parasit Vectors. 2015 Sep 30;8:498. doi: 10.1186/s13071-015-1108-7 (PMC4588682; doi:10.1186/s13071-015-1108-7)
Supplement: Additional file 1: Table S1. — The multistage antigen segments selected from T .gondii. Antigen fragments derived from tachyzoites, bradyzoies and sprozoites antigens of T. gondii were screened based on their predicted binding affinity to HLA (HLA-A*02, HLA-A*03 and HLA-B*07) and H2 (H2-Ld, H2-Dd and H2-Kd) supertype molecules. The epitopes from the antigen segments which have the percentile rank of lower than 50. Peptides (p1 to p7) marked in bold were derived from above protein fragments with high binding affinity and were used for analysis. (DOC 54 kb) [file 13071_2015_1108_MOESM1_ESM.doc]

# Table S1: The multi-stage antigen segments selected from *T .gondii*

| **Antigen^a^** | **Sequence^b^** | **Epitope^c^** | **MHC restriction^d^** |
| --- | --- | --- | --- |
| SAG3_101-144_ | GLGGEFLPLGGTSSYPRVCHIDAKDKGDCERNKGFLTDYIPGA | LPLEGATSS | H-2-Ld, HLA-B*07 |
|  |  | SSYPRVCHI | H-2-Kd,H-2-Ld, HLA-A*02 |
|  |  | EGATSSYPR | H-2-Dd |
|  |  | GATSSYPRV | HLA-A*02 |
|  |  | FLTDYIPGA | HLA-A*03, HLA-A*02, H-2-Dd, |
|  |  | YPRVCHIDA | HLA-B*07,H-2-Ld |
|  |  | **GLGGEFLPL(p1)** | H-2-Ld, Kd,Dd,HLA-A*02,A*03,B*07 |
| ROP18_347-396_ | RWVPNYFLLMMRAETDMSKVISWVFGDASVNNSELGLVVRMYLSSQAIRL | VPNYFLLMM | H-2-Ld, HLA-B*07 |
|  |  | YFLLMMRAE | H-2-Kd |
|  |  | WVPNYFLLM | H-2-Dd, HLA-A*02 |
|  |  | FLLMMRAET | HLA-A*02, H-2-Kd, |
|  |  | YLSSQAIRL | HLA-A*03 |
|  |  | SVNNSELGL | HLA-B*07, H-2-Kd |
|  |  | **RMYLSSQAI(p2)** | H-2-Ld, Kd,Dd,HLA-A*02,A*03,B*07 |
| MIC6_288-347_ | AGAIAGGVIGGLLLLSAAGAGVAYMRKSGSGGGEEIEYERGIEAAEASEVEVLVDLDSKT | AAGAGVAYM | H-2-Ld, HLA-B*07 |
|  |  | GVIGGLLLL | H-2-Kd, H-2-Ld, HLA-A*02 |
|  |  | AGAIAGGVI | H-2-Dd, H-2-Kd, HLA-B*07 |
|  |  | VLVDLDSKT | HLA-A*02 |
|  |  | GAGVAYMRK | HLA-A*03 |
|  |  | SAAGAGVAY | HLA-B*07, HLA-A*03 |
|  |  | **LLSAAGAGV(p3)** | H-2-Ld, Kd,Dd,HLA-A*02,A*03,B*07 |
| GRA7_182-224_ | GTVLGFAALAAAAAFLGMGLTRTYRHFSPRKNRSRQPALEQEV | RQPALEQEV | H-2-Ld, H-2-Dd, HLA-A*02 |
|  |  | AFLGMGLTR | H-2-Kd |
|  |  | FSPRKNRSR | H-2-Dd |
|  |  | GTVLGFAAL | HLA-A*02, HLA-B*07 |
|  |  | RTYRHFSPR | HLA-A*03 |
|  |  | SPRKNRSRQ | HLA-B*07, H-2-Kd |
|  |  | **ALAAAAAFL(p4)** | H-2-Ld, Kd,Dd,HLA-A*02,A*03,B*07 |
| MAG1_58-125_ | LLPQDAVLYENSEDVAVPSDSASTPSYFHVESPSASVEAATGAVGEVVPDCEEQQEQGDTTLSDHDFH | LPQDAVLYE | H-2-Ld |
|  |  | SVEAATGAV | H-2-Kd, HLA-A*03, HLA-A*02 |
|  |  | DSASTPSYF | H-2-Dd, H-2-Ld |
|  |  | VLYENSEDV | HLA-A*02 |
|  |  | LLPQDAVLY | HLA-A*03, H-2-Dd |
|  |  | SPSASVEAA | HLA-B*07, H-2-K, H-2-Ld |
|  |  | **SASTPSYFH(p5)** | \| H-2-Ld, Kd,Dd,HLA-A*02,A*03,B*07 \| \| --- \| |
| BAG1_156-211_ | VIKGEKTSKEAEKVDDGKTKNILTERVSGYFARRFQLPSNYKPDGSAAMDNGVLR | KPDGISAAM | H-2-Ld, HLA-B*07 |
|  |  | ISAAMDNGV | H-2-Kd, HLA-A*02 |
|  |  | SGYFARRFQ | H-2-Dd, H-2-Kd, |
|  |  | ISAAMDNGV | HLA-A*02, H-2-Dd, H-2-Kd |
|  |  | RVSGYFARR | HLA-A*03, H-2-Dd |
|  |  | RVSGYFARR | HLA-B*07, H-2-Ld |
|  |  | **GYFARRFQL(p6)** | H-2-Ld,Kd,Dd,HLA-A*02,A*03,B*07 |
| SPA_142-200_ | VPSDKCGVHILVKAAPQAPVCSAQDHTLELQITAANSDTSFVCGGTFNVIKPANAAKVL | KPANAAKVL | H-2-Ld, H-2-Kd, HLA-B*07 |
|  |  | SFVCGGTFN | H-2-Kd |
|  |  | AANSDTSFV | H-2-Dd,Kd,Ld, HLA-A*02,-B*07 |
|  |  | ILVKAAPQA | HLA-A*02 |
|  |  | VIKPANAAK | HLA-A*03 |
|  |  | VPSDKCGVH | HLA-B*07 |
|  |  | **FVCGGTFNV(p7)** | H-2-Ld, Kd,Dd,HLA-A*02,A*03,B*07 |

# Notes:

# ^a^ Antigens fragments SAG3_101-144_, ROP18_347-396_, MIC6_288-347_, GRA7_182-224_, MAG1_58-125_, BAG1_156-211_ and SPA_142-200_ derived from tachyzoites, bradyzoies and sprozoites antigens of *T. gondii* were screened based on their predicted binding affinity to HLA (HLA-A*02, HLA-A*03 and HLA-B*07) and H2 (H2-Ld, H2-Dd and H2-Kd) supertype molecules .

# ^b^ The sequence of the seven antigen segments.

# ^c^ The epitopes from the antigen segments which have the percentile rank of lower than 50. Peptides (p1 to p7) marked in bold were derived from above protein fragments with high binding affinity were used for analyzed.

^d^ The MHC restriction of the predicted epitopes.
